# Does Maternal Stress Affect the Early Embryonic Microenvironment? Impact of Long-Term Cortisol Stimulation on the Oviduct Epithelium

**DOI:** 10.3390/ijms21020443

**Published:** 2020-01-10

**Authors:** Shuaizhi Du, Nares Trakooljul, Jennifer Schoen, Shuai Chen

**Affiliations:** 1 Institute of Reproductive Biology, Leibniz Institute for Farm Animal Biology (FBN), Wilhelm-Stahl-Allee 2, 18196 Dummerstorf, Germany; du@fbn-dummerstorf.de; 2 Institute of Genome Biology, Leibniz Institute for Farm Animal Biology (FBN), Wilhelm-Stahl-Allee 2, 18196 Dummerstorf, Germany; trakooljul@fbn-dummerstorf.de

**Keywords:** stress, cortisol, preimplantation period, oviduct, air–liquid interface

## Abstract

Maternal stress before or during the sensitive preimplantation phase is associated with reproduction failure. Upon real or perceived threat, glucocorticoids (classic stress hormones) as cortisol are synthesized. The earliest “microenvironment” of the embryo consists of the oviduct epithelium and the oviductal fluid generated via the epithelial barrier. However, to date, the direct effects of cortisol on the oviduct are largely unknown. In the present study, we used a compartmentalized in vitro system to test the hypothesis that a prolonged stimulation with cortisol modifies the physiology of the oviduct epithelium. Porcine oviduct epithelial cells were differentiated at the air–liquid interface and basolaterally stimulated with physiological levels of cortisol representing moderate and severe stress for 21 days. Epithelium structure, transepithelial bioelectric properties, and gene expression were assessed. Furthermore, the distribution and metabolism of cortisol was examined. The polarized oviduct epithelium converted basolateral cortisol to cortisone and thereby reduced the amount of bioactive cortisol reaching the apical compartment. However, extended cortisol stimulation affected its barrier function and the expression of genes involved in hormone signaling and immune response. We conclude that continuing maternal stress with long-term elevated cortisol levels may alter the early embryonic environment by modification of basic oviductal functions.

## 1. Introduction

The beginning of life takes place in the oviduct, which is also called the “fallopian tube”. Upon successful sperm–oocyte recognition, fertilized embryos reside a few days within the oviduct developing (depending on the species) mostly up to the morula or even blastocyst stage before entering the uterus. The “microenvironment” of the early embryo consists of the oviduct epithelium, and the oviductal fluid generated via active epithelium secretion as well as passive and active transport across the epithelial barrier. This microenvironment guarantees optimal temperature, oxygen tension, PH, and nutrients to ensure embryo survival [1,2]. These early embryonic stages are considered a “sensitive window”, as alterations in environmental conditions have profound consequences on embryo development and an individual’s health in later life [3].

If a mammalian organism’s homeostasis is under real or perceived threat [4], the hypothalamic–pituitary–adrenal (HPA) axis is activated to synthesize glucocorticoids (GCs), i.e., classic stress hormones, in order to cope with the challenges. It has long been recognized that stress and elevated GCs disrupt reproduction and fertility at multiple levels [5,6]. In contrast to the well-characterized central interference of the HPA axis with the hypothalamic–pituitary–gonadal (HPG) system [6,7], the local actions of GCs in the female reproductive tract (FRT) remain largely unclear except for a few reports on ovaries [8].

Recent literature has reported in vivo and in vitro evidence that preimplantation maternal stress, specifically during the period when the embryo is hosted and transported by the oviduct, is closely associated with infertility and reproduction failure. In sows, stress evoked by food deprivation or repeated injection of adrenocorticotropic hormone (ACTH) during the postovulatory stage has been observed to lead to delayed ova transport, aberrant oviductal activity [9,10,11], endocrine profile changes, and impaired embryonic development [11]. Pregnant mice suffering from restraint stress during the embryonic oviduct transport period, as measured by elevated peripheral corticosterone levels (main rodent GC), have shown reduced embryo quality, conception rate, and litter size; furthermore, the negative consequences in this case were extended to behavior and physiology in postnatal life [12,13,14,15]. In vitro studies which exclude actions through the nervous system have revealed that zygotes co-cultured with mouse oviductal epithelial cells treated with high doses of corticosterone or corticotropin-releasing hormone (CRH) show reduced developmental competence (decreased blastocyst rate and blastocysts with decreased numbers of blastomeres) [16], while direct corticosterone/CRH exposure to mouse zygotes does not compromise embryo development. This altogether suggests that the effect of maternal GC on early embryos might be transmitted indirectly by the oviduct [16].

However, to date, almost no information is available on the short- or long-term effects of cortisol on the functionality of the oviduct epithelium. The pig shares some similarities in physiology, genetics, and metabolism with humans. Furthermore, pig sample materials are easily available as they are used for meat production; therefore, the pig is becoming increasingly popular as a model organism in biomedical research [17,18]. Especially for the study of female reproduction, in comparison to rodents, the pig is more analogous to humans in terms of hormonal status and estrous cycle length, although the pig has an estrous cycle and humans have a menstrual cycle [19]. We know that stressors affect the reproductive performance of female pigs during early pregnancy and that sows are especially susceptible to sustained elevation of cortisol and long-term stress [20,21]. Recently, we established a compartmentalized air–liquid interface (ALI) culture procedure which allows long-term culture of differentiated porcine oviduct epithelial cells (ALI-POEC) [22,23]. The cells are grown on porous filter supports which separate apical and basolateral compartments. The formed tissue-like epithelial layer actively produces an oviductal fluid surrogate on its apical side, while effectors (e.g., hormones or metabolites) can be administrated from the basolateral cell surface, mimicking in vivo conditions [24,25].

In the present study, we use this compartmentalized long-term culture system to test the hypothesis that long-term elevation of cortisol influences the early embryonic microenvironment by modifying the physiology of the oviduct epithelium. Hence, ALI-POEC were initially maintained to reach full differentiation. Then, physiological levels of cortisol representing moderate and severe stress were administrated basolaterally for a prolonged period of 21 days. We assessed the effect on epithelium structure, transepithelial bioelectric properties, gene expression related to oviduct functionality, and inflammation. Furthermore, the extracellular environment was examined from both apical and basal compartments for markers of cell damage, distribution, and metabolism of cortisol and cortisone.

## 2. Results

### 2.1. Effect of Long-Term Cortisol Stimulation on Morphology of ALI-POEC

**Figure 1:**
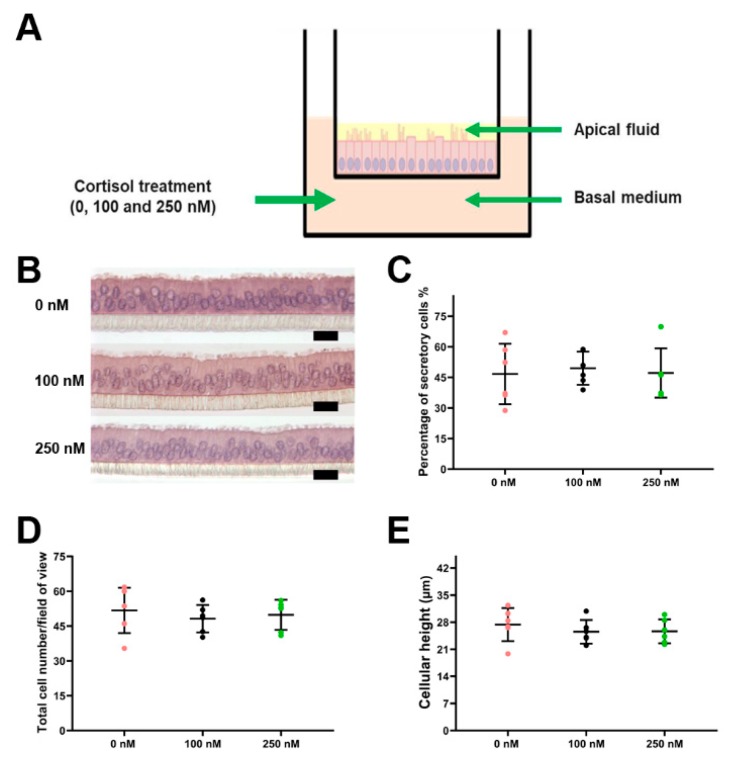
ALI-POEC morphology in response to long-term cortisol (100 and 250 nM) stimulation. (**A**) Schematic illustration of cortisol treatment in porcine oviduct epithelial cells grown at the air–liquid interface (ALI-POEC). (**B**) Representative cross-sections of ALI-POEC, hematoxylin–eosin (HE) staining, scale bar = 20 µm; (**C**) percentage of secretory cells; (**D**) total cell number/field of view; (**E**) cellular height. Data are shown as mean with standard deviation (SD). *n* = six animals.

The stimulation of the ALI-POEC system by basolateral administration of cortisol is illustrated in Figure 1A. Long-term exposure to cortisol caused no obvious effects on the morphology of ALI-POEC. Upon stimulation with 100 and 250 nM cortisol for 21 days, cells exhibited highly polarized structure, columnar shape, presence of cilia, and protrusions in a manner highly identical to the control cultures (0 nM, Figure 1B). Further quantification revealed that the percentages of secretory cells remained unaffected (*p* > 0.05; Figure 1C). Neither the total cell numbers (*p* > 0.05; Figure 1D) nor cellular height showed any significant changes by any level of cortisol treatment (*p* > 0.05; Figure 1E).

### 2.2. Long-Term Cortisol Stimulation Triggers the Canonical Glucocorticoid Receptor (GR) Pathway

**Figure 2:**
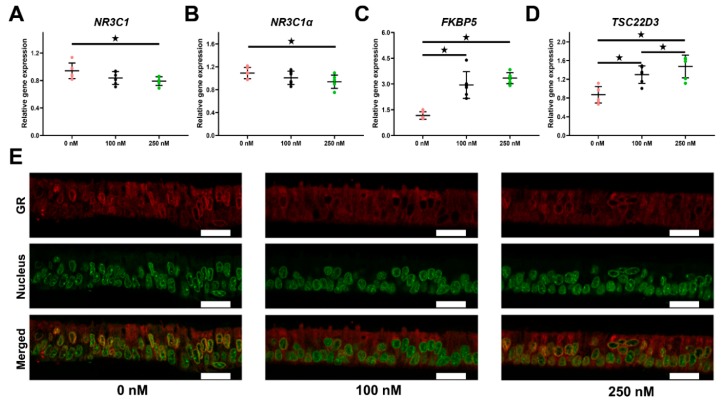
Activation of the glucocorticoid receptor (GR)-signaling pathway by cortisol in ALI-POEC. Differential marker gene expression of (**A**) *NR3C1*, (**B**) *NR3C1α*, (**C**) *FKBP5*, and (**D**) *TSC22D3*. Data are shown as mean with SD. Asterisks indicate a significant difference at *p* < 0.05. *n* = six animals. (**E**) Immunofluorescence staining of GR (red fluorescence) in ALI-POEC, nuclei stained with SYBR Green I; scale bar = 20 µm.

The mRNA expression of *NR3C1* (encoding GR proteins) and its dominating subtype *NR3C1*α were both found to be significantly down-regulated after 21 days of treatment with 250 nM cortisol (*p* < 0.05, Figure 2A,B). The transcriptional levels of FK506 binding protein 51 (*FKBP5*), a co-regulator of the GR signaling pathway, as well as TSC22 domain family member 3 (*TSC22D3*), a typical cortisol-inducible gene, were strongly elevated by cortisol in a dose-dependent manner (*p* < 0.05, Figure 2C,B).

The localization of GR protein was visualized by immunofluorescence. The results revealed that GR was mainly centered around the nucleus in the control group, which, however, became less evident upon cortisol stimulation (Figure 2E). Moreover, the fluorescence signal of GR protein was stronger in the control than the treated groups (Figure 2E), which was in line with the mRNA expression.

### 2.3. Long-Term Cortisol Treatment Alters Oviductal Functionality

#### 2.3.1. Transepithelial Bioelectric Properties

**Figure 3:**
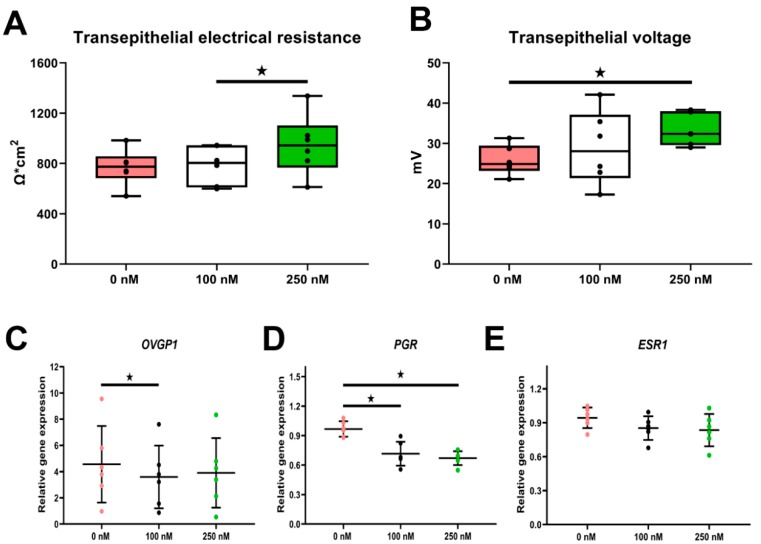
Effect of cortisol stimulation on oviductal functionality parameters in ALI-POEC. Elevation of transepithelial electrical resistance TEER (**A**) and transepithelial voltage (**B**); relative mRNA abundance of (**C**) oviduct-specific glycoprotein 1 (*OVGP1*), (**D**) progesterone receptor (*PGR*), and (**E**) estrogen receptor 1 (*ESR1*). Data are shown as mean with SD. Asterisks indicate a significant difference at *p* < 0.05. *n* = six animals.

To assess the barrier function and ionic transport of the oviduct epithelial layer, transepithelial electrical resistance (TEER) and transepithelial voltage assessments were carried out. All samples developed proper TEER falling into the range of good quality cultures [26], reflecting full confluence and differentiation of the epithelial layer (Figure 3A). Stimulation with 250 nM cortisol significantly increased the electrical resistance in comparison to the 100 nM group (*p* < 0.05, Figure 3A). Likewise, the transepithelial voltage was also significantly elevated in the 250 nM cortisol group (*p* < 0.05, Figure 3B).

#### 2.3.2. Long-Term Cortisol Stimulation Down-Regulated Expression of Oviductal Marker Genes

We further quantified the expression of key oviduct functional genes, including steroid hormone receptors. Oviduct-specific glycoprotein 1 (*OVGP1*) was significantly decreased by cortisol treatment (*p* < 0.05, Figure 3C). Similarly, both levels of cortisol remarkably down-regulated the transcription of progesterone receptor (*PGR*, *p* < 0.05, Figure 3D), while the expression of estrogen receptor 1 (*ESR1*) was not affected (*p* > 0.05, Figure 3E).

### 2.4. Impact of Long-Term Cortisol on Inflammation and Apoptosis

#### 2.4.1. Expression of Genes Related to Inflammation

**Figure 4:**
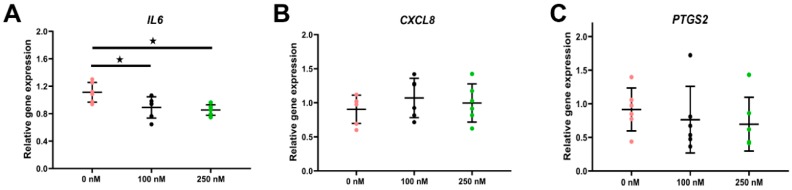
Effect of cortisol on inflammatory marker gene expression in ALI-POEC. Relative mRNA abundance of (**A**) *IL6*, (**B**) C-X-C motif chemokine ligand 8 (*CXCL8*), and (**C**) prostaglandin-endoperoxide synthase 2 (*PTGS2*). Data are shown as mean with SD. Asterisks indicate a significant difference at *p* < 0.05. *n* = six animals.

Considering the immunosuppressive effect of cortisol in vivo, we assessed the expression of immune-related genes after long-term cortisol stimulation. The pro-inflammatory cytokine *IL6* was significantly down-regulated by both cortisol doses (*p* < 0.05, Figure 4A). No regulation on C-X-C motif chemokine ligand 8 (*CXCL8*) or prostaglandin-endoperoxide synthase 2 (*PTGS2*) was observed (*p* > 0.05, Figure 4B,C).

#### 2.4.2. Long-Term Cortisol Treatment Does Not Trigger Apoptosis in ALI-POEC

**Figure 5:**
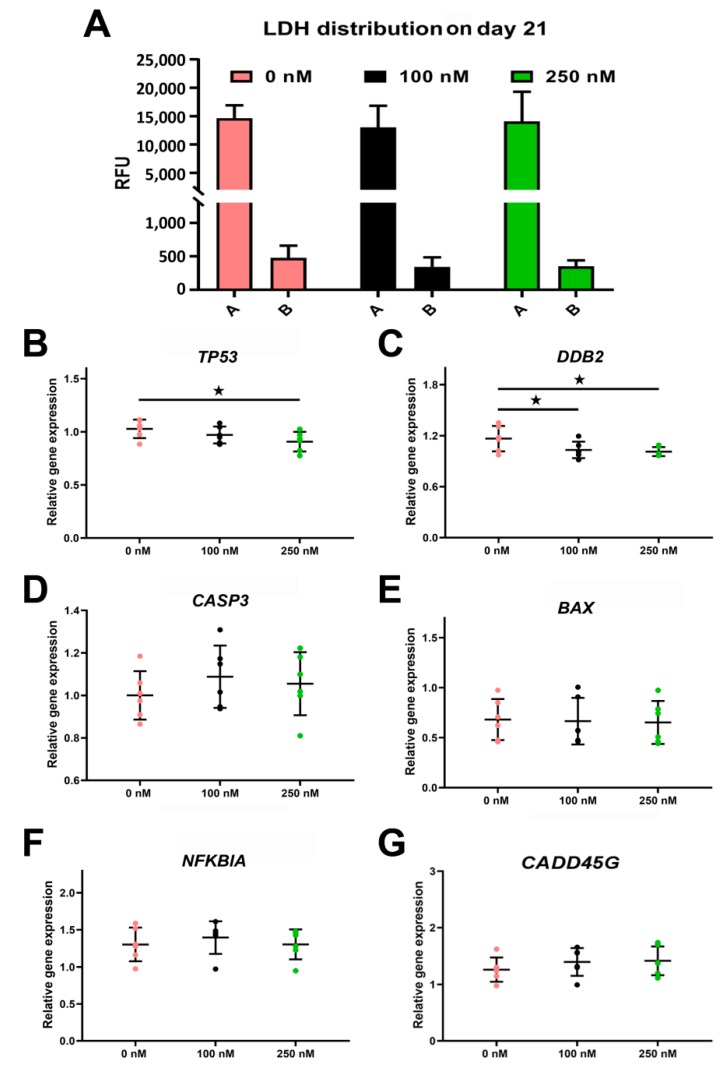
Apoptosis biomarkers in response to cortisol stimulation in ALI-POEC. (**A**) Constant lactate dehydrogenase (LDH) activity in apical and basal compartment. (**B**–**G**) Relative mRNA abundance of cell death marker genes. Data are shown as mean with SD. Asterisks indicate a significant difference at *p* < 0.05. *n* = six animals. Legend: RFU, relative fluorescence units.

We measured lactate dehydrogenase (LDH) activity in the apical and basolateral compartments of ALI-POEC. Although the cells had been exposed to cortisol for a prolonged period of 21 days, the release of LDH protein into both compartments revealed no significant differences (*p* > 0.05, Figure 5A). The LDH signal in the apical compartment, in general, was stronger than the basal compartment (Figure 5A).

The expression of genes related to DNA damage (*GADD45G*, Figure 5G) and cell death (*CASP3*, *BAX*, and *NFKBIA*) did not differ between the control and cortisol-treated groups (Figure 5D–F). Cortisol stimulation slightly suppressed the expression of *TP53* and *DDB2* (Figure 5B,C).

### 2.5. Distribution and Metabolism of Cortisol and Cortisone in the ALI-POEC System

**Figure 6:**
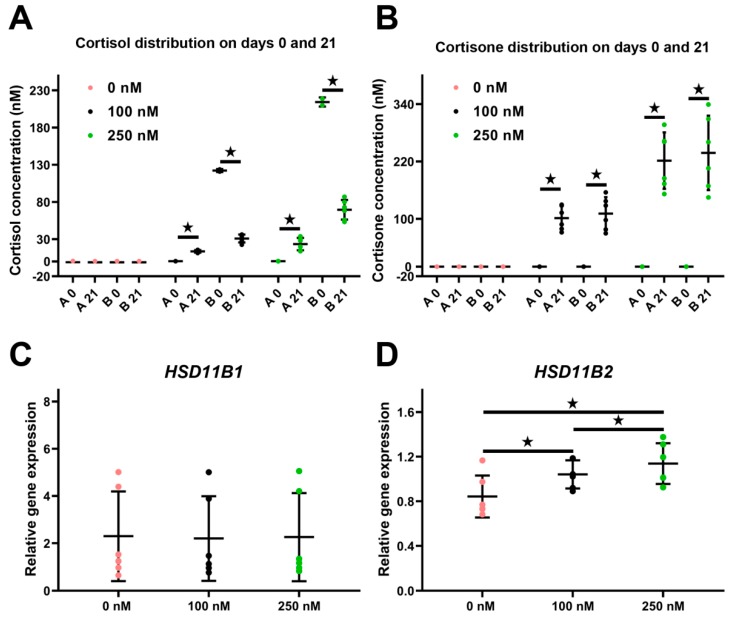
Distribution and metabolism of cortisol and cortisone in ALI-POEC. (**A**) Directional changes of cortisol distribution during treatment; (**B**) rising cortisone levels in apical and basal compartment. Relative mRNA abundance of (**C**) *HSD11B1* converting cortisone to active cortisol and (**D**) *HSD11B2* inactivating cortisol to cortisone. Data are shown as mean with SD. Asterisks indicate a significant difference at *p* < 0.05. *n* = six animals.

In the control group, cortisol and cortisone were detected neither in the apical fluid nor in the basal medium from day 0 to 21 (Figure 1A and Figure 6A,B). On day 0, 122.22 ± 2.04 nM and 222.38 ± 12.31 nM cortisol were detected in the basal medium of cultures treated with 100 nM and 250 nM cortisol, respectively (Figure 6A), while cortisone remained undetectable (Figure 6B). On day 21 (12 h after the last cortisol application), cortisol concentration in the basal medium was 30.79 ± 5.45 nM (100 nM group) and 69.51 ± 11.26 (250 nM group). Cortisol in the apical fluid increased to 13.61 ± 1.36 nM (100 nM group) and 23.34 ± 7.38 nM (250 nM group), respectively, on day 21. The cortisol level in the medium (basal cell side) remained higher than in the oviductal fluid surrogate on the apical side of ALI-POEC (Figure 6A). Inversely, on day 21, the cortisone level grew evidently in both the apical fluid and basal medium, reaching 101.68 ± 25.14 nM (apical) and 110.92 ± 34.47 nM (basal) in the 100 nM group and 221.79 ± 59.02 nM (apical) and 238.07 ± 77.79 nM (basal) in the 250 nM group (Figure 6B).

The expression of hydroxysteroid 11-beta dehydrogenase 1 (*HSD11B1*) and 2 (*HSD11B2*), the enzymes converting cortisone to cortisol and vice versa, were assessed by RT-qPCR. Cortisol treatment did not change the expression of *HSD11B1* but did induce a significant dose-dependent upregulation of *HSD11B2* (Figure 6C,D).

## 3. Discussion

Activation of the stress axis during early pregnancy disrupts fertilization and early embryo development inter alia via elevated cortisol [27,28]. However, the direct local actions of cortisol at the upper FRT are largely unknown. In this study, we initially applied the ALI-POEC culture system to investigate the effect of sustained cortisol elevation on the early embryonic microenvironment. The applied compartmentalized culture system holds serval unique features. It faithfully remodels the structure and functionality of oviduct epithelium tissue in vivo. It allows stimulation of the epithelium from the basolateral cell pole, therefore better simulating the cortisol supply from the arterial vascular bed. The compartmentalized system furthermore permits simultaneous monitoring of physiological situations, in both the apical (corresponding to the oviductal lumen) and basal (corresponding to sub-epithelial maternal tissue) compartments. As the culture system is applicable for long-term culture, we were able to mimic a prolonged stress period of 21 days without cell passaging or any sign of cell dedifferentiation.

In general, upon the 21 days of cortisol administration, neither the dose that mimics moderate stress (100 nM) nor the one simulating severe stress (250 nM) provoked any obvious alternation to the gross morphology of the in vitro oviductal epithelium, as revealed by histomorphometry. Cells maintained their fully differentiated status with constant composition of ciliated and secretory subtypes, though a slight but not significant decline in cell numbers and epithelium height was noticed. However, TEER, a highly sensitive parameter used to assess epithelium integrity that is largely determined by tight junctions [26], showed a significant increase in electrical resistance in the “severe stress” group. In line with our findings, Zhaeentan et al. have recently reported that cortisol treatment in human fallopian tube epithelial cells regulates the expression of tight junction genes [29]. Similarly, a TEER increase after cortisol stimulation has been observed in an intestinal epithelial cell line (Caco-2/Bbe cells) [30]. The movement of charged ions across the epithelium layer is essential for oviductal fluid formation [31]. Active transport of solutes like chloride (Cl^−^) and sodium (Na^+^) produces a potential difference (PD), which could be measured as transepithelial voltage. We found that long-term cortisol stimulation leads to a marked increase in PD, suggesting enhanced unidirectional trafficking of certain ions across the oviductal epithelium. Collectively, this indicates an effect of long-term cortisol stimulation on the oviductal barrier function.

Additionally, the expression of oviductal marker gene *OVGP1* and hormone receptors was altered by cortisol stimulation. *OVGP1*, which plays multi-functional roles in sperm–zona pellucida binding, fertilization, early embryo cleavage, and development [32], was notably suppressed. It is well known that oviduct functionality is regulated by ovarian-derived hormones, namely estrogen (E2) and progesterone (P4), which fluctuate during the estrous cycle [33]. The level of *ESR1* remained unaffected, whereas expression of *PGR* was markedly down-regulated by both moderate and high levels of cortisol, hinting at a profound impairment of the oviduct epithelium responsiveness to P4 after a long-term cortisol challenge.

Major cortisol action takes place through the activation of the GR (encoded by the *NR3C1* gene). Hence, we assessed the effect of long-term cortisol stimulation on the classical GR signaling pathway. Long-term cortisol treatment slightly down-regulated the mRNA of the main subtype *NR3C1*α in oviductal cells. Expression of the GR inducible gene *FKBP5* sharply increased in a dose-dependent manner, which could adversely modulate GR signaling by causing less efficient nuclear translocation of the receptor complex [34]. Likewise, *TSC22D3* (an indicator of GR pathway sensitivity) was also strongly up-regulated in a dose-dependent manner [35]. Recent evidence suggests that GCs hold both anti- and pro-inflammatory effects related to the complex mechanisms of GR signal transduction [36].

Cortisol is well known for its immune suppressive functions. In luminal epithelial cells of the bovine endometrium cortisol down-regulated mRNA expression of pro-inflammatory cytokines, like *IL6* and *CXCL8* [37]. Accordingly, long-term cortisol stimulation also suppressed IL6 expression in ALI-POEC. However, *CXCL8* expression was not affected, pointing at an oviduct specific pattern of pro-inflammatory cytokine regulation.

Previous studies in mice have reported that preimplantation restraint stress triggers apoptosis in oviducts, thereby leading to embryo mortality [14]. In our study, we quantified the amount of LDH, a cytoplasmic enzyme released to the cell environment when the plasma membrane is damaged during apoptosis, necrosis, and other forms of cellular damage, in both the apical secretion and basolateral medium after long-term cortisol stimulation [38]. Surprisingly, our results revealed that the amount of dead or damaged cells was not affected on either side, which was further supported by the expression of apoptosis markers, e.g., *TP53*, *CASP3*, and others. This finding is inconsistent with the in vivo study. We hypothesize that apoptosis is not caused by direct cortisol action on the oviduct epithelium but may be rather regulated by the HPG axis, which was not mimicked in our culture approach.

There are not many studies available which report cortisol content within the oviductal fluid. The cortisol levels measured in ALI-POEC apical fluid after long-term cortisol stimulation are roughly in accordance with recent data from bovine oviductal fluid [39]. The oviductal cortisol levels measured in this in vivo study were surprisingly high compared to the physiological plasma cortisol levels in cattle. However, the oviductal fluid was gained from slaughterhouse by-products and so probably from animals highly stressed by transport and slaughter.

The ALI-POEC system revealed the massive cortisol metabolizing capacity of the oviduct epithelium. Even after three weeks of repeated cortisol application, the majority of bioactive cortisol in the culture system was converted to its inactive metabolite cortisone. Thus, the epithelial cells actively prevented high cortisol concentrations in the apical fluid. In line with this finding, long-term cortisol stimulation led to an upregulation of *HSD11B2* mRNA expression, the enzyme that converts cortisol to cortisone, in the oviduct epithelium.

In conclusion, we investigated the effect of maternal stress on the early embryonic microenvironment by long-term stimulating highly differentiated oviduct epithelial cells with cortisol concentrations measured in vivo during moderate and severe stress. The oviduct epithelium was able to metabolize cortisol and thereby stabilize the cortisol concentration reaching the embryonic microenvironment. However, extended cortisol stimulation affected the barrier function and marker gene expression of the in vitro oviduct epithelial tissue. The expression of genes involved in the hormone responsiveness and immunological functions of the oviduct were impaired. This supports our hypothesis that long-lasting maternal stress associated with long-term elevated cortisol levels may affect the early environment of the embryo by modification of basic oviductal functions. However, in this study we only analyzed the long-term effects of cortisol in a rather static system (cortisol application every 12 h). The time-dependent adaptation of ALI-POEC to the cortisol stimulus as well as the effect of steady cortisol concentrations (e.g., via perfusion of the culture system) needs further investigation. Finally, embryo co-culture experiments will prove if and how cortisol affects embryonic development via the luminal epithelial tissue.

## 4. Materials and Methods

### 4.1. Media and Reagents

DMEM/Ham’s F-12, fetal bovine serum (FBS), 4-(2-Hydroxyethyl)piperazine-1-ethanesulfonic acid (HEPES), and amphotericin B were purchased from Merck Millipore (Billerica, MA, USA). Other reagents were obtained from Sigma Aldrich (St. Louis, MO, USA) unless otherwise indicated.

### 4.2. Tissue Collection and Cell Isolation

The collection of porcine tissue and cell isolation was performed following our previously published protocol [23,40]. Briefly, porcine oviducts of healthy gilts (approximately six months old) were collected from a local slaughterhouse (Danish Crown Teterower Fleisch GmbH, Teterow, Germany) where these animals were slaughtered for meat production purposes. Only oviducts of non-cycling gilts were included in the study. The oviduct epithelial cells were isolated by digestion with collagenase 1A and accutase. Isolated cells were seeded on hanging inserts or cryopreserved in liquid nitrogen for long-term storage.

### 4.3. ALI-POEC Culture and Cortisol Stimulation Experiments

#### 4.3.1. ALI-POEC Culture

ALI-POEC cultures using cells from individual animals (from *n* = six donors, one 24- and one 12-well insert/group/animal) were carried out as recently described by our group [23] with slight modifications. From day 0 to day 6, cells were cultured in proliferation-inducing medium (M1) under liquid–liquid interface condition at 37 °C, 5% CO_2_, and 5% O_2_. After day 7, the cells were switched to ALI condition using serum-free medium (M2a) which contained neither cortisol nor cortisone. Medium change and apical fluid removal were performed twice a week. After three weeks of culture, cells were used for cortisol stimulation.

#### 4.3.2. Cortisol Preparation and Stimulation

Two concentrations of cortisol (100 and 250 nM), as measured in the plasma of sows under moderate and severe stressors, were employed for the stimulation experiment [41,42]. The 10 mg/mL cortisol stock solution was prepared in 100% ethanol and stored in aliquots at −20 °C until use. Serum-free M2a medium [23] with the desired cortisol concentrations (0, 100, or 250 nM) was administrated to the basolateral compartment of ALI-POEC for a period of 21 days (Figure 1A). Our pretest revealed that cortisol levels in the medium of ALI-POEC dramatically dropped after 12 h. Thus, for the purpose of mimicking chronic stress over a long time period with a sustained high level of cortisol, the stimulation medium was refreshed at 12 h intervals. Accordingly, the medium in the control group, which contained solvent only, was also changed every 12 h.

### 4.4. TEER and Transepithelial Voltage Assessment

Before harvesting the 24-well ALI culture, TEER and transepithelial voltage were measured using an EVOM2 Epithelial Voltohmmeter (WPI, Sarasota, FL, USA). To minimize any offset, the electrodes were equilibrated by soaking them in 100 mM KCl for 24 h before measurement. The apical compartment of the ALI culture system was refreshed with 200 µL of preequilibrated DMEM/Ham’s F-12, and then the ohmic resistance and voltage of samples along with a blank sample (culture insert without cells) were measured within 5 min to ensure a sustained temperature. The final unit area resistance (Ω*cm^2^) and transepithelial voltage were calculated per the manufacturer’s instructions.

### 4.5. Determination of Cortisol, Cortisone, and LDH in the Apical and Basal Compartments

Previously, we have reported that ALI-POEC produces an oviductal fluid surrogate in the apical compartment [23]. Generally, within 3 days cells cultured in a 24- or 12-well insert are able to produce approximately 10–30 µL fluid in the apical compartment. A timespan of at least 3 days is therefore needed to collect and analyze components of the apical fluid. Therefore, for the cortisol/cortisone and LDH assay, on day 18 of cortisol stimulation, the apical side of cells was gently rinsed three times with prewarmed DMEM/Ham’s F-12 to remove any dead cells or debris. As mentioned in Section 4.3.2, the stimulation medium in the basolateral side was changed every 12 h. On day 21 of stimulation, apical fluid (generated from day 18–21) and basal medium (12 h after medium change) were both recovered (see Figure 1A) and centrifuged at 13,000 × *g* at 4 °C for 10 min, after which supernatants were collected and stored at −70 °C until further use.

The concentrations of cortisol were assessed by an enzyme-linked immunosorbent assay (ELISA) kit (Enzo Life Sciences Inc., New York, USA; ADI-900-071), while levels of cortisone were measured using a commercially available chemiluminescent immunoassay (CLI) kit (Abbor Assays, Ann Arbor, MI, USA; K017-C1). The fluorometric LDH activity assay kit (Abcam, Boston, MA, USA; ab197004) was used to determine cellular cytotoxicity. For all assays, samples were measured in duplicate.

A FLUOstar Omega microplate reader (BMG LABTECH GmbH, Ortenberg, BW, Germany) was used for the optical density (wavelength at 405 nm for cortisol), luminescence (for cortisone), and fluorescence (excitation/emission = 535/587 nm for LDH) reading within 5 min after plate preparation. The concentrations of hormones were calculated against the corresponding standard curves prepared in the same matrix, utilizing BMG Omega Mars software (BMG LABTECH, Ortenberg, BW, Germany). Relative fluorescence units (RFU), which directly correlate to the number of damaged cells, estimated the LDH activity.

### 4.6. Histomorphometry and Immunofluorescence

The histological processing followed our previously described procedure [25]. Paraplast embedded samples were cut into 4 µm sections for hematoxylin–eosin (HE) or immunofluorescence staining. For histomorphometric analysis, HE stained images (5× images/sample) were taken at 400× magnification to measure the cellular height (5× positions/image), total cell number, and the number of secretory cells using the ImageJ software (National Institutes of Health, Bethesda, MD, USA) [43].

Immunofluorescence staining was performed to identify the subcellular localization of the GR protein. For antigen retrieval, sections were immersed and cooked in 100 mM citrate buffer for 3 min. Sections were blocked with 3% BSA for 30 min at RT and incubated with anti-GR antibody (Abcam; ab3578; 1:200) overnight at 4 °C. Antigen was detected by Alexa Fluor 647 conjugated polyclonal goat anti-rabbit antibody (Thermo Fisher Scientific, Dreieich, HE, Germany; A-21245; 1:200) and the nucleus was counterstained with SYBR Green I (Thermo Fisher Scientific, Dreieich, HE, Germany; S-7563; 1:200). The slides were imaged at 400× magnification by a confocal laser scanning microscope LSM 800 equipped with Zen software (Carl Zeiss, Oberkochen, BW, Germany).

### 4.7. Gene Expression Analysis

Gene expression of ALI-POEC was quantified by RT-qPCR as recently reported [33]. All primer sequences, annealing temperatures and PCR efficiencies are listed in Table S1. Briefly, total RNA was prepared using the NucleoSpin RNA kit (Macherey-Nagel, Dueren, NW, Germany) and quantified using NanoDrop ND-1000 (Thermo Fisher Scientific, Dreieich, HE, Germany). ALI-POEC of the six donor animals (biological replicates) were used in each treatment group. cDNA was synthesized from 1 µg of total mRNA by RevertAid reverse transcriptase (Thermo Fisher Scientific, Dreieich, HE, Germany). Each qPCR analysis was performed in duplicate (for the six biological replicates) using SensiFast™ SYBR No-ROX reagents (Bioline Reagent, Ltd., London, UK) and a LightCycler 96 system (Roche, Mannheim, BW, Germany).

The threshold cycle (CT) value was automatically determined for each reaction with the analysis software LightCycler 96 (Roche). For each primer pair, standard qPCR analysis was performed to determine the CT values for a 10-fold dilution series of the cDNA template. Calibrated amplification curves were generated to evaluate the PCR efficiency. Specificity of the RT-qPCR reactions was determined by melting curve analysis and sequencing of the amplified products.

The CT values were then converted into relative quantities in comparison to one randomly chosen control sample using the 2^−ΔΔCT^ method and corrected by the corresponding PCR efficiency.

The stability of four reference genes, including beta-actin (*ACTB*), succinate dehydrogenase complex flavoprotein subunit A (*SDHA*), glyceraldehyde-3-phosphate dehydrogenase (*GAPDH*), and transforming growth factor β-stimulated clone 22 domain family member 2 (*TSC22D2*) were determined using the geNorm algorithm [44]. The normalization factor was generated based on the geometric mean of the two most stable endogenous reference genes (*TSC22D2* and *SDHA*).

### 4.8. Statistical Analysis

Data were analyzed by SPSS Statistics 25 for Windows (IBM Corp., Armonk, NY, USA). The normality of the data was tested by the Schapiro-Wilk method. Data were analyzed by repeated-measures analysis of variance (ANOVA) followed by post hoc comparisons with the Fisher least significant difference (LSD) test. For data that did not follow a normal distribution, the Friedman rank sum test was conducted, followed by the Wilcoxon rank sum test with LSD correction. In all experiments, *p* < 0.05 was considered as significant.

## Supplementary Materials

Supplementary materials can be found at https://www.mdpi.com/1422-0067/21/2/443/s1.

## Abbreviations

HPA	Hypothalamic–pituitary–adrenal
GCs	Glucocorticoids
HPG	Hypothalamic–pituitary–gonadal
ACTH	Adrenocorticotropic hormone
CRH	Corticotropin-releasing hormone
ALI	Air–liquid interface
ALI-POEC	Porcine oviduct epithelial cells grown at the air–liquid interface
GR	Glucocorticoid receptor
TEER	Transepithelial electrical resistance
LDH	Lactate dehydrogenase
