# Supplementary material for: Does Maternal Stress Affect the Early Embryonic Microenvironment? Impact of Long-Term Cortisol Stimulation on the Oviduct Epithelium
Source: Int J Mol Sci. 2020 Jan 10;21(2):443. doi: 10.3390/ijms21020443 (PMC7014231; doi:10.3390/ijms21020443)
Supplement: Supplementary file 1 [file ijms-21-00443-s001.pdf]

Table S1. Primer sequences used for RT-qPCR

Gene symbol	Forward Primer (5′ to 3′)	Reverse Primer (5′ to 3′)	Fragment size (bp)	Tm (ºC)	PCR efficiency
TSC22D2	AGACTCAGACCCAGCCTTTG	CAACAGGAGGCTTCACAACA	120	60	1.99
ACTB	CAACTGGGACGACATGGAG	GAGTCCATCACGATGCCAG	234	60	1.92
GAPDH	ATTCCACCCACGGCAAGTTC	AAGGGGCAGAGATGATGACC	225	60	1.96
SDHA	CTACAAGGGGCAGGTTCTGA	AAGACAACGAGGTCCAGGAG	141	60	1.94
NR3C1	CACCTGGATGACCAAATGACC	AGGGTAAAGCCATTCTCTGCTC	151	60	1.98
NR3C1α	TCAGAACTGGCAACGCTTTTATCAAC	AATGTCTGGAAGCAATAGTTAAGGAG	93	60	1.98
FKBP5	TGCCATTTACTGTGCAAACCAG	TTCTTCGGATAATGCCTCCATC	148	63	1.97
TSC22D3	GCAGGCCATGGATCTGGTG	GGGCTCGCCAGGGTCTTC	144	60	1.99
OVGP1	GGGGCACTTTCTGTGGCACT	AGCCAGGCTTTCAGGGCAAG	149	60	1.96
PGR	TGAGAGCACTAGATGCCGTTGCT	AGAACTCGAAGTGTCGGGTTTGGT	197	60	1.98
ESR1	AGGGAAGCTCCTGTTTGCTCC	CGGTGGATATGGTCCTTCTCT	234	60	1.90
HSD11B1	TTCTCAACCACATCACCCAC	AGTTGACCTCCATGCTTCTG	81	60	1.98
HSD11B2	GGTCAAGGTCAGCGTCATC	CAAGTGCTCGATGTAGTCCTC	148	60	1.96
IL6	ATAAGGGAAATGTCGAGGCTG	GTGGCTTTGTCTGGATTCTTTC	88	60	1.98
CXCL8	GCTCTCTGTGAGGCTGCAGTT	TTTATGCACTGGCATCGAAGTT	62	60	1.96
PTGS2	AGAGCTTCCCGATTCAAAGG	CCTCGCTTCTGATCTGTCTTG	144	60	1.94
TP53	GGAACAGCTTTGAGGTGCGTGTTT	ATACTCGCCATCCAGTGGCTTCTT	182	60	1.96
DDB2	GATTCGGGTTTACTCTGCCTC	AAATTAGGATCTGGGTATCGGC	150	60	1.97
CASP3	ATTGGACTGTGGGATTGAGAC	ACCAGGTGCTGTAGAATATGC	106	60	1.95
BAX	GCTGACGGCAACTTCAACTG	GCGTCCCAAAGTAGGAGAGG	202	60	1.96
NFKBIA	TGTCTTTGGGTGCTGATGTC	CTGGTAGGTGACTCTGTTGAC	140	60	1.95
GADD45G	ACTCTGGAAGAAGTTCGCGG	TTGTCGGGGTCCACATTCAG	164	60	1.95
